# Age, Technology, and the Digital Divide: Are They Directly Related to Mental Health Problems?

**DOI:** 10.3390/healthcare12232454

**Published:** 2024-12-05

**Authors:** Marina Barreda Gutiérrez, David Cantarero-Prieto, Marta Pascual Sáez

**Affiliations:** Health Economics Research Group—Biomedical Research Institute Marques de Valdecilla (IDIVAL), Santander Financial Institute (SANFI), University of Cantabria—Foundation UCEIF, 39005 Santander, Spain; david.cantarero@unican.es (D.C.-P.); marta.pascual@unican.es (M.P.S.)

**Keywords:** digital divide, older adults, DID, SHARE, mental health

## Abstract

In current times, we coexist with technology, using it every day. However, in older people, the use and employability of technology on a day-to-day basis is often more complicated or even null due to a lack of knowledge. Background/Objectives: The youngest generation were born surrounded by technology, which has given them superior capabilities when it comes to handling technology compared to elderly people. In short, older people have grown up without technology and only later in life have they crossed paths with it. Therefore, these circumstances can produce what is known as a “digital divide”, an unequal distribution in the access, use, or impact of information and communication technologies among social groups. Thus, the aim of this study is to examine whether there is a digital divide among European older adults and to show its effect on the mental health of individuals. In this way, we analyze how technological characteristics (digital divide) cause worsen mental health. Additionally, we examine whether, over time, the digital divide has had a greater impact on the mental health of older adults. Methods: For this purpose, recently published data from the Survey on Health, Aging, and Retirement in Europe are used. Results: Our analysis has revealed that the digital divide, driven by age, negatively affects the mental health of older adults in Europe. Thus, we have analyzed how technological characteristics related to the digital divide lead to poorer mental health among this population. Additionally, we have examined whether the digital divide has increasingly impacted older adults’ mental health over time. Conclusions: These findings highlight the need to address the digital divide as a public health issue, promoting greater digital inclusion to improve the psychological well-being of older adults.

## 1. Introduction

The evolution and development of the technologies in our lives is undeniable. Furthermore, the benefits they offer have made people’s lives in modern society more comfortable. This has led to what is known as an increase in welfare statistics, driven by the produced social developments.

The increase in well-being is primarily due to the application of Information and Communication Technologies (ICT) in individuals’ daily lives. ICT has had an increasingly strong influence on society over the past 70 years, especially following the industrial revolutions [1]. This development has resulted in various advantages, such as the incorporation of ICT into different aspects of our lives by governments and other institutions. These applications include health management, the development of social services, immediate access to information, the breakdown of distance barriers between people, enhanced productivity and efficiency, and the simplification of tasks [2].

Similarly, the increase in life expectancy has led to rapid growth in the elderly population in many European countries. The World Health Organization (WHO) (2022) [3] forecasts that the proportion of the global population aged 60 and over will double between 2015 and 2050. However, despite the benefits of ICT, many older adults struggle to adapt to new technologies. European and national surveys show widespread ICT use, with approximately 99% of the European population surveyed reporting Internet use within the last three months [4]. In Spain, 92.9% of individuals aged 16–74 were frequent Internet users in 2022 [5]. In short, these statistical results demonstrate that technology is now ubiquitous, with the highest percentages of frequent Internet users found among young people. As age increases, however, the frequency of Internet use decreases for both men and women.

The term “digital divide” is often used to refer to the difficulties people face in accessing ICT due to economic inequalities. However, the concept, as defined by the OECD in 2001 [6], refers more broadly to the gap between individuals, households, companies, and geographic areas at different socioeconomic levels in terms of access to and use of ICT. As a result, the digital divide reveals significant disparities both between and within countries. Studies [7] have highlighted the existence of different levels of the digital divide. The level we aim to examine in this study focuses on the social inequality created by age. It is crucial to understand why older people, even those with positive attitudes towards technology, use ICT less frequently, in order to help them navigate social changes and close the digital divide.

The rapid pace of technological evolution and the aging population have intensified the digital divide, an inequality in the access to and use of ICT that disproportionately affects older adults. This difficulty in adapting to the digital environment not only limits access to essential services but is also associated with negative effects on mental health and well-being [8,9]. Despite the advantages of ICT, studies show significant generational differences in their usage, emphasizing the need to address the digital divide as a public health issue.

The objective of this study is to determine and examine how ICT has evolved and its impact on the mental health of older adults over time. We begin with the premise that ICT have made significant progress to become firmly integrated into daily life as we know it today. Consequently, we analyze whether the lack of ICT management or skills, due to the digital divide created by age, contributes to mental health problems.

The structure of this study is as follows: In Section 2, we present the state of the art through a literature review. In Section 3, we describe the methodology and data used for this study. In Section 4, we report the results obtained from various regression analyses. Finally, in Section 5, we discuss the results and draw conclusions.

## 2. Literature Review

The group of interest in our study is the elderly, defined as individuals aged 55 and older. While older adults are generally considered to be those aged 60 or older [10], this study focuses on individuals aged 55 and above. This decision is based on data availability and the nature of the group being examined, as individuals aged 55 were not raised in a technological environment but have had to adapt to and learn how to use technology throughout their lives. This approach allows us to analyze the generational transition in ICT use from a perspective that better reflects the actual experience of adaptation that these individuals have undergone [11].

Older age groups face difficulties with ICT [12], which is why many authors refer to it as a digital divide [13]. This could be due to factors such as poor usability or a diminished ability to learn due to the age cohort [14]. Therefore, identifying sociodemographic characteristics that are associated with greater usability can help to improve the effectiveness of eHealth interventions and reduce the digital divide.

Many authors identify factors that negatively influence the digital divide. For instance, there is a digital divide between rural residents, older adults, and low-income groups [15]. Some studies consider age and educational level as key determinants of the digital divide [9]. Other research [16] has found that advancing age and living in assisted facilities negatively affect digital access. In fact, individuals with psychotic disorders and functional impairments often have much less access to digital technology compared to the general population. Additionally, recent evidence [12] shows that older adults demonstrate a positive relationship between self-assessed computer skills and mental health, cognitive abilities, and physical health. Furthermore, factors such as having a partner, education level, and self-rated writing skills have been found to be the best predictors of computer skill levels in older adults. In terms of mental health, other studies have demonstrated that gerontechnology anxiety affects ICT use and self-efficacy among older adults in rural areas of Korea [17]. Additionally, other authors [18] have shown that older adults from lower-income groups are more likely to suffer from the digital divide.

In summary, the evidence shows that digitalization, particularly the establishment of a digital lifestyle within the target group, presents challenges that contribute to the digital divide.

## 3. Data and Methodology

### 3.1. Data Sample

The data used for the analyses in this study come from the Survey of Health, Aging and Retirement in Europe (SHARE), a longitudinal survey that includes information on more than 120,000 people aged 50 and older across 27 European countries plus Israel. SHARE consists of various modules that collect data on household characteristics, sociodemographic variables, health status, lifestyle factors, cognitive impairment, mental health, social support, and the use of both health and non-health resources.

The analysis period covers the years 2013 (wave 5), 2015 (wave 6), 2017 (wave 7), and 2019/20 (wave 8). Given the objectives of the study, we selected subjects from all European countries with follow-up data from different waves. This approach covers both the period of evolution and development of digital life (waves 5, 6, and 7) and the period in which digital life is considered fully established in daily life (wave 8).

The selection of waves 5 and onward is particularly relevant, as these are the first waves in which information on technological skills was included in the SHARE survey. These waves correspond to a period of significant evolution and development in the establishment of digital life as we know it today, marking a transitional phase in which the integration of digital technologies into daily routines began to become more widespread. Waves 5, 6, and 7 thus provide a critical window for examining how older adults adapted to the increasing presence of digital tools and services during a time of rapid technological advancement. In addition, wave 8 represents a point at which digital life is considered fully integrated into everyday life, with the use of digital technologies reaching near-universal adoption in daily activities. For instance, a notable example is the difference in mobile phone usage between the periods corresponding to waves 5, 6, and 7, compared to wave 8, where there is clear evidence of the full consolidation of mobile technologies in everyday life [19].

The original sample size of the European respondents in the selected waves of the SHARE database is approximately 65,733 observations. However, after selecting observations with relevant information, appropriate variables, and valid values for individuals, the sample size is reduced to a smaller number of observations.

### 3.2. Variable Selection

The dependent variable in this study is “MentalHealth”, which measures mental health problems. To define this variable, we collected data from a questionnaire that asked whether the individual had experienced any mental health issues. The results for this question are expressed using the EURO-D depression scale, a short scale to measure symptoms of depression [20]. However, the scale does not specify what is considered a “normal” level of symptoms. For all individuals, the points on the scale do not have the same significance when it comes to defining good or bad mental health, or even good or bad health in general. This is where the concept of normality in health comes into play. According to previous definitions, normality is based on assessing the absence of symptoms, meaning that the presence of symptoms indicates abnormality [21]. Therefore, the fundamental issue with this criterion is that there are no fixed or absolute symptoms of abnormality.

To determine whether individuals have mental health problems, we rely on the existing literature. Previous studies, such as those by [22,23], identify the optimal cut-off point for poor mental health as a score of 4 or higher on the EURO-D scale, based on the “DSM-IV” and depression criteria from “GMS/AGECAT”. Both studies also show the internal consistency and validity of the EURO-D scale in the European Survey of Health, Aging, and Retirement in Europe. Consequently, in our study, we define individuals as having mental health problems if their score on the EURO-D scale is greater than or equal to 4.

Moreover, the dependent variable was categorized as binary: 1 if the respondent suffers from poor mental health (EURO-D score of 4 or higher) and 0 if otherwise.

Other variables were also considered in relation to the digital divide and mental health. These included age, gender, education level (no education, low, medium, and high according to ISCED-97 codes), digital ability, number of children, and area of residence. The age groups in this study were divided into three categories: 55–64 years, 65–80 years, and over 80 years. This segmentation reflects a common distinction in studies of aging and digital skills. People aged 55–64 are generally in a pre-retirement or early retirement phase, during which they are still professionally active and maintain regular contact with technology. In contrast, those aged 65–80 have entered active retirement and may begin to experience changes in their relationship with technology, though they still have substantial access to digital devices. The over-80 age group, on the other hand, often faces greater physical and cognitive limitations that affect their interaction with technology. Several studies, including those by [24,25], have shown that the ability to interact with digital technologies changes significantly with age, especially when comparing the 65–80 age group with those over 80. These studies emphasize the need to segment these age ranges to better understand the differences in access, knowledge, and adoption of digital technologies across generations.

Regarding digital ability, the variable created to measure digital skills includes various elements that provide a more comprehensive picture of individuals’ technological competencies. Several variables were combined to form a final variable that captures different aspects of digital ability. This approach was necessary because the SHARE survey does not include a single variable that encompasses all aspects of digital skills. Computer use and frequent Internet use are two key but distinct components of digital competence. While computer skills are important, technology today involves more than just computers. Access to and the frequent use of the Internet are equally relevant, as they are closely linked to digital skills beyond basic computer handling. Since there is no single variable that covers all of these aspects, we combined responses related to computer use and Internet frequency to form a final variable that more accurately reflects respondents’ digital skills. This combination allows for a more complete measurement of technological competencies, as today, individuals need not only the ability to use digital devices but also the ability to navigate and utilize the information available on the Internet. This method provides a more holistic and realistic measure of digital skills, taking into account the most relevant technological tools in people’s daily lives [26].

The coding of variables was designed to simplify the analysis, following methods used in previous studies. This approach facilitates the clearer interpretation and comparison of results. Future research could explore more detailed distinctions, but for this study, this method provides an effective way to address the key factors influencing mental health in older adults and the digital divide.

A detailed description of the variables included in the analysis can be found in Table 1.

### 3.3. Statistic Analysis

In this study, to evaluate the effect of managing and using ICT on mental health, we apply a differences-in-differences (DID) approach. 

The DID method is designed to estimate effects or causal relationships by identifying a specific intervention or treatment, and then comparing the differences in outcomes before and after the treatment [27]. Among the existing studies, one of the most well-known applications of the DID method is the article by [28] on the impact of the minimum wage in New Jersey. This demonstrates that DID is an established and widely used technique in studies that compare changes over time and analyze the effects of policies.

For the purposes of this study, we aim to estimate the differential effect of a treatment in the “treatment group” versus the “control group” over two periods: before the “treatment”, which involves individuals who do not use or lack ICT skills, and after the “treatment”, which involves individuals who have ICT skills. Specifically, we seek to determine whether there is an incremental effect on mental health problems stemming from the ability to manage and use ICT.

Therefore, thanks to the DID, the effect of specific treatments can be calculated. This is how [29] defined it:

(1) $${ATE}_{1}=E\left(w=1\right)=E\left(w=1\right)-E(Y_{0}|w=1)$$

where Y_0_ and Y_1_ represent mental health problems for people who have some ability with ICT and those who do not.

Specifically, the DID estimator relies on strong identification assumptions [30]. As such, potential violations of these assumptions should be taken into account. One such assumption is the presence of common trends, which may not hold in the context of ICT usability. To address this, we consider data from the time periods in which we wish to disentangle the impact of ICT usability. By comparing the treatment group before and after the treatment, we can assess periods when ICT was less important and still developing versus a period in which digital life became fully integrated into everyday life.

To avoid potential issues with time series data, such as trends, the DID method is the most appropriate. By controlling for covariates, we can infer two key fixed effects: the first related to digital life (λ), and the second related to ICT usability and/or ability (δ), along with the interaction between these two factors (γ). According to [31], the estimator in the context of OLS regressions can be derived as follows:

(2) $${MentalHealth}_{it}=\alpha +\delta {ability}_{it}+\lambda t_{i}+\gamma \left(ability*t\right)+\beta^{\prime }X_{it}+\epsilon_{it}$$

where ${MentalHealth}_{it}$ denotes the mental health of individual i at time t; ${ability}_{it}$ represents a dichotomous variable on the level of usability and/or ability of individual i at time t; $t_{i}$ is the time point; and $X_{it}$ denotes a set of individual features. $\epsilon_{it}$ is the error term.

Model 1 includes time dummies, a dummy variable for ICT skills and usability, and the interaction between time dummies and ICT skills and usability. The set of sociodemographic characteristics in Model 1 includes age and sex. To account for the influence of other covariates, we expanded Model 1 in Model 2 by adding the education level and whether the individual has children. Finally, in Model 3, we further controlled for an additional life condition: whether the individual resides in a rural area.

To clarify the application of the DID method in this study, it is essential to specify the treatment and control groups. The treatment group consists of individuals who have ICT skills and use these technologies, while the control group is made up of individuals who do not have ICT skills and do not use ICT. The key distinction between these groups is analyzed over two time periods: one before ICT became fully integrated into daily life (when the treatment group had less interaction with technology) and another after it had become a regular part of daily routines (comparing 2019 vs. 2013, 2019 vs. 2015, and 2019 vs. 2017).

The use of the DID method in this study is justified by its ability to estimate the causal effects of “ICT skills and use” on mental health, while controlling for common trends that might affect both groups over time. The goal is to compare the evolution of mental health problems between the treatment and control groups before and after the period when ICT became embedded in daily life. Since ICT did not have the same level of relevance or accessibility in the past, the DID method allows us to isolate the specific impact of ICT skills and use on participants’ mental health, controlling for potential external factors that could similarly influence both groups.

Thus, the DID methodology aligns well with the objectives of this study, enabling a more accurate assessment of the differential impact of digital skills on mental health without interference from pre-existing trends that could otherwise distort the results.

## 4. Results

### 4.1. Summary Statistics

Table 2 presents the summary statistics of the sample for the set of covariates included in the analysis for each wave. The proportion of individuals experiencing mental health problems increases over time. In 2013, 27.2% of respondents reported mental health issues. This proportion grew gradually, reaching 29.9% in 2019/20. Thus, as technology continues to develop and becomes more integrated into daily life, the number of older adults facing mental health challenges also rises. However, it is worth noting that in 2017 (wave 7), the proportion of individuals with mental health problems slightly decreased compared to 2015 (wave 6). Despite this, the percentage rose again in the following wave.

The vast majority of the variables analyzed follow similar and consistent trends. Regarding the variable that measures the ability to use and manage ICT among older adults, we observed a decline in ICT ability over time, particularly from Wave 5 to Wave 7. This decline is gradual, with an increasing inequality in ICT skills as time progresses. However, it is important to note that in Wave 8, there is a slight recovery in ICT ability, although it remains lower than in Wave 5. Specifically, the proportion of respondents who considered themselves to have technology skills was higher in 2013 (53.5%) than in 2019/20 (50.5%), but still lower than in Wave 5.

The gender variable shows an upward trend, with the proportion of women consistently higher than that of men across all waves. In the last wave (2019/20), approximately 62% of the respondents with these characteristics were women.

In terms of education, very few individuals were without education across Waves 5, 6, 7, and 8, with the proportion remaining at around 0.04%. For those with low and medium levels of education, the proportions are higher, generally ranging between 35% and 40%. The proportion of individuals with higher education is lower, hovering around 20%.

The variable indicating whether respondents have children follows a constant trend, gradually increasing over time. In all waves, around 90% of those surveyed reported having children. As for the area of residence, the trend for individuals living in rural areas shows only slight variation. Approximately 30% of respondents in each wave reported living in rural areas.

Finally, we believe it is important to highlight the results regarding respondents’ ability to use and manage ICT, broken down by age. As shown in Table 3, the proportion of individuals who report difficulties with ICT increases with age. This indicates the existence of a digital divide caused by age.

### 4.2. Regression Results

The estimates of the DID results on skills and technology management are presented in Table 4. It is important to note that the coefficients of the models correspond to the specifications in Equation (2). While the parameter of primary interest is γ, which measures the change in the effect of mental health in relation to the ability and/or management of technologies compared to 2013, 2015, and 2017, other results are also reported. Specifically, the parameter δ captures the change in each of the variables based on the respondents’ technology skills. The parameter λ, on the other hand, accounts for the effects of time periods on the development of technology skills over time. The results table is shown below.

As shown in Table 4, in all the time periods that are compared to obtain results, the interaction term is statistically significant. This means that there is an effect regarding the ability and/or management of mental health technologies over time among European older adults. Specifically, it is found that the negative effect of not having sufficient ICT management skills on mental health problems intensifies over time and the older you are. The establishment of digital life for the elderly is a significant issue.

Throughout the models, different variables are shown within the vector X of covariates that are related to the results obtained (changes given the effect of ICT ability in 2019/20 on mental health compared to 2013, 2015, and 2017). The variables that are related throughout the models are age, gender, education, children, and area of residence.

The interaction term “γ” is positive and significant for all mental health models, suggesting that the negative effects of not having skills in technologies on people’s mental health are intensified due to the evolution and establishment of technologies in the daily lives of individuals.

As can be seen in Table 4, all the variables (except in specific cases) have a significant impact on the mental health of the respondents. The variable that relates to age is statistically significant in all DID models, except in Model 3 when it compares the results of 2019 with 2017 for the age group of 65 to 80 years. The results of their regression show that at a younger age, the risk of mental health problems is lower. However, when it is observed that the respondents are older than 80, the results change, showing a greater risk of suffering from mental health problems.

In relation to gender, the results show that if you are a woman, the risk of suffering from mental health problems is greater. Regarding education, it is observed that educational levels do not generate greater risks of suffering from mental health problems, as is the case if you have children or live in a rural area.

When examining the impact of the area of residence (urban vs. rural) over time, it is evident that living in a rural area has a differential effect compared to urban living. Specifically, the variable “area of residence” is statistically significant across the years 2019−2013, 2019−2015, and 2019−2017, but with varying degrees of impact. In Model 1, the coefficient for rural areas shows that individuals living in rural areas are more likely to experience mental health problems, with this effect being consistent when comparing 2019 with 2013 and 2015. However, as we move to Model 2 and Model 3 (2019−2017 comparison), the significance becomes weaker, suggesting that the impact of living in a rural area on mental health may have diminished slightly over time, especially when accounting for the increasing integration of digital life into daily routines.

In terms of the variable “children”, the results show that having children is statistically significant for mental health in all models, but the effect changes slightly depending on the time comparison. From 2013 to 2019, and from 2015 to 2019, respondents with children exhibit a stronger protective effect against mental health issues, with a coefficient consistently above 0.7. This suggests that the presence of children can act as a buffer against the negative effects of ICT skills and mental health over time. However, in the 2017−2019 comparison, the coefficient for having children (0.951) remains significant but slightly weaker, suggesting that the effect of children as a protective factor has diminished somewhat over the years, perhaps due to the greater challenges of digital adaptation faced by older adults in more recent years.

In summary, the differences in area of residence and the impact of having children on mental health outcomes between 2013, 2015, and 2017 to 2019 show significant variations. While living in rural areas appears to have a consistent negative impact on mental health in earlier years, this effect seems to be diminishing as technology becomes more accessible. Similarly, while having children initially showed a stronger protective effect against mental health problems, this protective effect seems to weaken slightly in the most recent years, possibly due to the increasing complexity of managing ICT skills in later life.

## 5. Discussion

In this study, our objective was to evaluate how the evolution and integration of technology into the daily lives of older adults has impacted their mental health, specifically examining how digital life affects older populations using four waves of the SHARE survey. Our research contributes to the literature in several important ways.

Firstly, we compared data across different time periods, which allowed us to highlight variations in technology’s impact over time. This approach also exposes limitations in the survey, particularly the challenge of making extended time comparisons due to the gaps in the available data. Secondly, our analysis, which covers 27 European countries over seven years (2013–2019/20), provides a comprehensive view of how technology adoption has influenced mental health among older adults. The wide geographic scope and extended time frame enhance the robustness of our findings, offering insights that are both broad and deep.

The results show a significant relationship between the growth of technology use and an increased probability of mental health problems among older adults. This finding is critical for policymakers, as it underscores the wide-reaching consequences of technology on the well-being of elderly populations. It is clear that technology, while essential in modern society, has unintended negative consequences for older individuals who struggle to keep up. Therefore, it is essential to implement learning programs for older adults and to ensure that support staff are available where technology use is required. Appropriate policies must be designed to ensure that access to health services, social support, and information does not create further barriers for older people [32]. These policies should be flexible and adaptable, designed to meet the unique needs of aging populations.

Contrary to the assumption that technological advancements automatically improve societal well-being [33], our study demonstrated that older individuals’ inability to use technology is a significant trigger for adverse mental health outcomes. While technological development is generally viewed as beneficial, our findings highlight that it also creates challenges, especially for older adults who may be less familiar with these tools. The digital divide, exacerbated by age, was found to lead to mental health issues by 2019/20, with no substantial changes in its impact over the years. This suggests that the digital divide is not an issue that emerged during the COVID-19 pandemic but has been a longstanding problem that has only gained more attention in recent years.

Technology, instead of enhancing well-being, appears to place an additional burden on older adults [34], increasing their risk of mental health issues. This finding is significant because it points to the social divide created by age, as older adults struggle to adapt to and manage technological devices. Governments must recognize that while promoting technology is attractive from a policy standpoint, the unique needs of the aging population must be taken into account. Failure to do so could put a strain on national and European budgets, particularly given the increasing life expectancy across Europe. Moreover, age-related cognitive decline is a critical factor that further complicates the ability of older adults to interact with technology and may further exacerbate the challenges they face [35].

Although this study primarily focuses on European data, the findings may be extrapolated to Spain, which, according to the United Nations [36], is projected to have the second oldest population by 2050. Age has already been identified as a key factor affecting mental health, further emphasizing the relevance of our research in a country with a rapidly aging population [37]. However, it would be valuable to conduct additional studies in specific contexts, adapting the methodologies to local characteristics, and assessing whether the patterns observed here hold in other cultural and economic settings.

In summary, the digital divide caused by age has been widely documented by several authors, particularly during the COVID-19 era [38,39], when the rapid shift to digital solutions further highlighted the exclusion of older adults. However, this study shows that the digital divide was an issue long before the pandemic, affecting the mental health of older adults well before this period. This underscores that technological disconnection is not a new phenomenon but rather a longstanding issue that has gained more visibility in recent years.

Finally, this study has several limitations that should be acknowledged. First, the temporal scope of the data is limited. Although we used four waves of the SHARE survey (2013–2019/20), long-term comparisons are constrained by the lack of earlier or later data. This limitation prevents a broader analysis of trends or significant shifts in the relationship between technology use and mental health over extended periods. Second, the data used in the study are self-reported, which may introduce biases due to recall issues or differences in interpretation by respondents. Lastly, although the study covers 27 European countries, cultural and socioeconomic differences between these nations may affect how technology impacts mental health in different contexts.

## 6. Conclusions

In conclusion, our analysis reveals that the digital divide, driven by age, has a detrimental impact on the mental health of older adults in Europe. As the European population continues to age, it is essential for public policies to evolve in order to help older individuals adapt to digital technologies, ultimately ensuring better health outcomes and a more successful integration of digitalization. The magnitude of this issue will depend largely on whether governments take decisive action to realign their policies in ways that promote convergence in both health and technology, addressing the mental health challenges associated with digital inequality.

To make these policy recommendations more effective, it is critical to explore practical implementation strategies. One potential approach is the creation of “digital health hubs” in local communities, where older adults can receive hands-on training and ongoing support in using digital health tools and ICT. Additionally, governments could incentivize the development of user-friendly technologies tailored to the needs of older adults, such as simplified health apps or devices designed to improve cognitive and physical well-being. Partnerships with technology companies could also facilitate access to affordable devices and reliable internet services for older adults, particularly in underserved or rural areas, helping to bridge the digital divide.

Implementing these recommendations requires a multi-faceted approach that integrates education, technology, and mental health support. By focusing on these key areas, we can help older adults to better navigate the digital world, enhancing their ability to manage health conditions and improving their overall mental health.

Studies like this are critical for identifying the specific needs of older adults regarding both technology and mental health. Ongoing research in this area will ensure that policies remain responsive and effective in addressing these evolving challenges.

While this study makes important contributions, it also has several limitations that should be acknowledged. First, the inability to analyze earlier waves (pre-2013) due to data availability restricts the scope of our analysis and prevents a more comprehensive examination of long-term trends. Additionally, the use of self-reported data to assess mental health introduces subjectivity into the analysis, as the assessment of mental health depends on individuals’ perceptions of their own well-being, rather than a clinical diagnosis. This could introduce biases or inaccuracies, as individuals may not fully recognize or accurately report mental health challenges. Moreover, the variability in how mental health problems is understood and reported across different individuals complicates comparisons and could affect the consistency of the findings.

Another limitation is the omission of other potential factors that might influence both ICT skills and mental health, such as pre-existing health conditions, social support, or socioeconomic factors. These factors may interact with digital skills in ways that are not captured by our analysis. Furthermore, while the study focuses on the general relationship between ICT skills and mental health, it does not differentiate between the specific types of technologies used by older adults. The impact of different digital tools—such as smartphones, social media, or health-monitoring devices—may vary, and understanding these nuances would provide a more detailed picture of how technology affects mental health.

Additionally, while our study covers a broad European population of older adults, it does not account for regional or cultural differences that might shape how digital skills are developed or how they influence mental health outcomes. Given the diversity in technology access and usage across different European countries, the findings may not be universally applicable to all older populations. Lastly, the study’s cross-sectional nature limits its ability to establish causality. While the DID methodology helps to control for some confounding variables, it cannot fully account for all dynamic factors influencing both ICT use and mental health over time. Longitudinal studies, following individuals over extended periods, would be necessary to draw more definitive conclusions about causality.

## Figures and Tables

**Table 1:** List of variables and codes.

Variable	Label	Coding
Mental Health	Respondent’s mental health status	1: respondent has suffered from mental health problems; 0: otherwise.
Ability	Digital skills of the respondent	1: respondent has digital skills; 0: otherwise.
Education	Education Code ISCED-97	0: no education; 1: low educational level; 2: level of secondary education; 3: high level of education.
Area	Location area (residence)	1: respondent livesin a rural area; 0: otherwise.
Gender	Respondent gender	1: female; 0: male.
Age 50 to 64	Respondent’s age	1: the respondent’s age is between 50 and 64; 0: otherwise.
Age 65 to 80	Respondent’s age	1: the respondent’s age is between 65 and 80; 0: otherwise.
Age 80 plus	Respondent’s age	1: the respondent’s age is over 80 years; 0: otherwise.
Children	Number of children	1: respondent has one or more children; 0: otherwise.

Source: Own elaboration based on SHARE survey.

**Table 2:** Main statistics (mean).

	Wave 5 (N = 41,021)	Wave 6 (N = 41,713)	Wave 7 (N = 9307)	Wave 8 (N = 7258)
Mental Health	0.272	0.288	0.285	0.299
Ability	0.535	0.515	0.477	0.505
*Age*				
Age 50 to 65	58.511	58.856	62.684	62.839
Age 65 to 80	72.228	72.288	72.478	72.432
Age 80+	85.254	85.297	85.739	85.784
Gender	0.586	0.600	0.603	0.611
*Education*				
No Education	0.041	0.040	0.037	0.038
Low Education	0.353	0.355	0.420	0.380
Medium Education	0.372	0.380	0.336	0.340
High Education	0.234	0.225	0.207	0.231
Children	0.889	0.891	0.892	0.896
Area	0.311	0.323	0.321	0.285

Source: Authors’ elaboration. Reference categories: with technology skills, woman, with children and rural area.

**Table 3:** Proportion of people without ICT skills according to age.

	Wave 8	Wave 7	Wave 6	Wave 5
Age to 50–65	23.02	30.31	28.74	27.86
Age to 65–80	43.75	50.39	57.45	57.32
Age 80+	78.99	82.04	85.04	85.87

Source: Authors’ elaboration.

**Table 4:** Results of the analysis of differences-in-differences on mental health and the digital divide.

	2019–2013			2019–2015			2019–2017		
Variables	Coefficients Model 1	Coefficients Model 2	Coefficients Model 3	Coefficients Model 1	Coefficients Model 2	Coefficients Model 3	Coefficients Model 1	Coefficients Model 2	Coefficients Model 3
γ (Interaction)	2.296 ***	2.362 ***	2.355 ***	2.203 ***	2.295 ***	2.305 ***	2.258 ***	2.310 ***	2.326 ***
	(0.113)	(0.133)	(0.135)	(0.108)	(0.129)	(0.131)	(0.138)	(0.162)	(0.166)
δ (Ability)	0.490 ***	0.553 ***	0.553 ***	0.507 ***	0.552 ***	0.549 ***	0.487 ***	0.545 ***	0.536 ***
	(0.001)	(0.014)	(0.015)	(0.010)	(0.014)	(0.014)	(0.02)	(0.028)	(0.028)
λ (Year)	0.728 ***	0.730 ***	0.737 ***	0.707 ***	0.688 ***	0.694 ***	0.740 ***	0.732 ***	0.732 ***
	0.026	0.029	0.030	0.025	0.027	0.028	0.031	0.035	0.035
*Age*									
Age 65 to 80	0.943 ***	0.890 ***	0.894 ***	0.943 ***	0.916 ***	0.904 ***	1.033 ***	0.975 ***	0.97
	(0.019)	(0.025)	(0.022)	(0.018)	(0.021)	(0.022)	(0.042)	(0.047)	(0.047)
Age 80+	1.363 ***	1.212 ***	1.188 ***	1.335 ***	1.198 ***	1.183 ***	1.333 ***	1.232 ***	1.213 ***
	(0.038)	(0.04)	(0.04)	(0.035)	(0.038)	(0.039)	(0.066)	(0.071)	(0.071)
Gender	1.941 ***	1.935 ***	1.914 ***		1.960 ***	1.953 ***	1.989 ***	2.023 ***	2.009 ***
	(0.035)	(0.042)	(0.043)		(0.042)	(0.043)	(0.062)	(0.075)	(0.076)
*Education*									
Low Education		0.676 ***	0.670 ***		0.739 ***	0.740 ***		0.691 ***	0.684 ***
		(0.032)	(0.032)		(0.036)	(0.035)		(0.056)	(0.056)
Medium Education		0.530 ***	0.529 ***		0.589 ***	0.586 ***		0.561 ***	0.559 ***
		(0.026)	(0.026)		(0.029)	(0.029)		(0.048)	(0.047)
High Education		0.461 ***	0.457 ***		0.544 ***	0.538 ***		0.516 ***	0.519 ***
		(0.024)	(0.024)		(0.028)	(0.028)		(0.047)	(0.047)
Children		0.868 ***	0.870 ***		0.904 ***	0.900 ***		0.951	0.944
		(0.028)	(0.029)		(0.029)	(0.029)		(0.053)	(0.053)
Area			0.932 ***			0.958 *			1.058
			(0.021)			(0.021)			(0.049
N (Observations)	72.527	50.632	48.293	73.473	51.572	48.986	23.176	17.075	16.580
Log-pseudolikelihood	−40,171.221	−28,438.638	−27,144.114	−41,515.513	−29,715.596	−28,154.402	−13,241.078	−9890.16	−9598.3029
Prob > chi2	0.000 ***	0.000 ***	0.000 ***	0.000 ***	0.000 ***	0.000 ***	0.000 ***	0.000 ***	0.000 ***

Clustered standard errors at the individual level in parentheses. *** *p* < 0.01. Reference categories: with technology skills, from 50 to 65 years old, woman, without studies, with children, and rural area of residence.

## Data Availability

Data are contained within the article.
